# A Review of the Functional Annotations of Important Genes in the AHPND-Causing pVA1 Plasmid

**DOI:** 10.3390/microorganisms8070996

**Published:** 2020-07-03

**Authors:** Hao-Ching Wang, Shin-Jen Lin, Arpita Mohapatra, Ramya Kumar, Han-Ching Wang

**Affiliations:** 1 The Ph.D. Program for Translational Medicine, College of Medical Science and Technology, Taipei Medical University and Academia Sinica, Taipei 110, Taiwan; 2 Graduate Institute of Translational Medicine, College of Medical Science and Technology, Taipei Medical University, Taipei 110, Taiwan; arpitamohapatra6481@gmail.com; 3 International Center for the Scientific Development of Shrimp Aquaculture, National Cheng Kung University, Tainan 701, Taiwan; z10303066@email.ncku.edu.tw (S.-J.L.); ramyak12@gmail.com (R.K.); 4 Department of Biotechnology and Bioindustry Sciences, College of Bioscience and Biotechnology, National Cheng Kung University, Tainan 701, Taiwan; 5 Mits School of Biotechnology, Utkal University, Bhubaneswar, Odisha 751004, India

**Keywords:** acute hepatopancreatic necrosis disease, *Vibrio parahaemolyticus*, pVA1 plasmid, PirA*^vp^*, PirB*^vp^*, transposases, conjugation factors, antirestriction proteins, secretion system post-segregational killing system, DNA methyltransferase

## Abstract

Acute hepatopancreatic necrosis disease (AHPND) is a lethal shrimp disease. The pathogenic agent of this disease is a special *Vibrio parahaemolyticus* strain that contains a pVA1 plasmid. The protein products of two toxin genes in pVA1, *pirA^vp^ and pirB^vp^*, targeted the shrimp’s hepatopancreatic cells and were identified as the major virulence factors. However, in addition to *pirA^vp^ and pirB^vp^*, pVA1 also contains about ~90 other open-reading frames (ORFs), which may encode functional proteins. NCBI BLASTp annotations of the functional roles of 40 pVA1 genes reveal transposases, conjugation factors, and antirestriction proteins that are involved in horizontal gene transfer, plasmid transmission, and maintenance, as well as components of type II and III secretion systems that may facilitate the toxic effects of pVA1-containing *Vibrio* spp. There is also evidence of a post-segregational killing (PSK) system that would ensure that only pVA1 plasmid-containing bacteria could survive after segregation. Here, in this review, we assess the functional importance of these pVA1 genes and consider those which might be worthy of further study.

## 1. Introduction

Acute hepatopancreatic necrosis disease (AHPND) is a bacterial disease that causes severe damage in shrimp farming [1]. In 2009, the first outbreak of AHPND was seen in China, and the disease quickly spread to Vietnam, Malaysia, Thailand, Philippines [1], and Bangladesh [2]. According to a recent report, it is now also present in the USA [3].

The original AHPND-causing bacteria was identified as a specific strain of *Vibrio parahaemolyticus* [4,5,6,7,8,9,10]. *V. parahaemolyticus* is a Gram-negative halophilic bacterium that can be found in marine environments [11]. In the past, *V. parahaemolyticus* was already recognized as a causal agent for human acute gastroenteritis in raw, contaminated sea foods [11], and it contains two hemolysin virulence factors, thermostable direct hemolysin (*tdh*), and TDH-related hemolysin (*trh*), that are both confirmed to cause damage to human cells [11]. Now, however, by acquiring a unique plasmid, pVA1, that encodes the binary pore-forming toxins PirA*^vp^* and PirB*^vp^* (often written as simply PirAB*^vp^*), *V. parahaemolyticus* has also become a lethal pathogen to penaeid shrimps [4,5,6,7,8,9,10]. The major clinical AHPND symptom in *V. parahaemolyticus*-infected penaeid shrimps is a pale-to-white atrophied hepatopancreas (HP) [4,5,6,7,8,9,10]. Histological examinations further showed that PirAB*^vp^* toxins caused shrimp HP cell death and led to the characteristic sloughing of the damaged epithelial cells into the HP Tubules [4,5,12].

Although the binary PirAB*^vp^* toxins together seem to play the main role in damaging the HP cells [5,9,10], the pathogenesis of AHPND-causing *V. parahaemolyticus* is not yet fully understood. For example, we still do not know how *V. parahaemolyticus* colonizes the various shrimp organs, or how do shrimp cells respond to *V. parahaemolyticus* infection. In the course of the disease, apart from the PirAB*^vp^* toxins, there is also very little that is known of the functional roles of other genes/proteins from the pVA1 plasmid, as well as those from the *V. parahaemolyticus* bacterium itself. To address these issues, advanced techniques, such as next generation sequencing, structural biology, and system biology have been applied [5,13,14]. These approaches have led to a recent report by Kumar et al., which found that crude bile acids affect the AHPND-causing *V. parahaemolyticus* by inducing biofilm formation and triggering an increased release of PirAB*^vp^* toxins [14]. In addition, transcriptomic analysis of AHPND-causing *V. parahaemolyticus* treated with crude bile acid showed alterations in several metabolic and cellular signaling pathways [14]. However, although studies that look beyond the PirAB*^vp^* toxins can provide valuable insights into AHPND, to date, there are still only a handful of reports that relate to other gene products of the pVA1 plasmid.

## 2. The AHPND-Causing Plasmid pVA1

**Figure 1:**
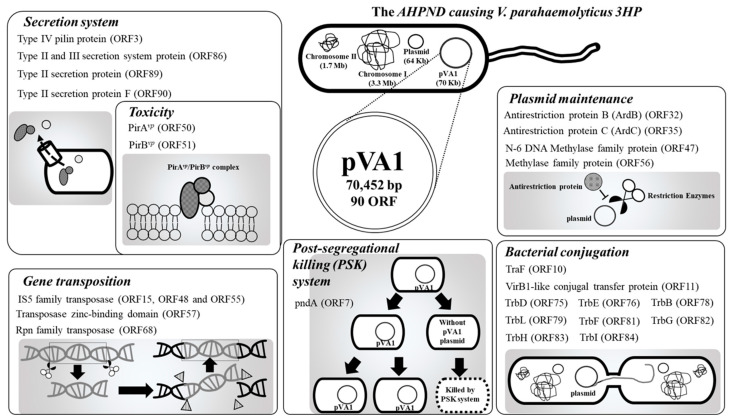
Functional classifications of the pVA1 genes discussed in this review. All information in this figure (e.g., open-reading frames (ORF) number) refers to the plasmid pVA1 of the *V. parahaemolyticus* strain 3HP (NCBI accession number: KP324996).

The AHPND-causing plasmid, pVA1, was first discovered by next-generation sequencing (NGS). By comparing the DNA sequences between *V. parahaemolyticus* strains that caused AHPND (3HP, 5HP, and China) and those that did not (S02), a 69-kb plasmid, pVA1, was found in the 3HP, 5HP, and China strains but not in the S02 strain [15,16,17,18,19]. Subsequently, the pVA1-encoded binary pore-forming PirAB*^vp^* toxins were identified as the key factors in the AHPND pathology [5], and in addition to specific strains of *V. parahaemolyticus*, other *Vibrio* species, such as *V. harveyi*, *V. campbellii*, *V. owensii*, and *V. punensis,* have also become AHPND-causing agents [20,21,22,23]. All of these AHPND-causing *Vibrio* spp. harbor pVA1-related plasmids that contain the *pirA* and *pirB* toxin genes [5,20,21,22,23]. Recently, a non-*Vibrio* bacterium, *Microccocus luteus*, was also reported to carry the *pirA* and *pirB* toxin genes [24]. Another report confirmed that pVA1 *pirAB^vp^* genes could be transferred from an AHPND-causing *V. parahaemolyticus* to a non-*Vibrio* and non-pathogenic *Algoriphagus* sp. [25]. These developments suggest that pVA1-related plasmids could be transmitted between different species, which would make it more difficult to control AHPND. It is already known that pVA1-related plasmids contain a number of genes for horizontal gene transfer, plasmid transmission, and maintenance, including the post-segregational killing (PSK) system. Meanwhile, components of type II and III secretion systems may further facilitate the toxic effects of pVA1-containing bacterial spp. Therefore, in addition to the toxic *pirA* and *pirB* genes, these plasmid-related genes (Figure 1; Table 1) are also worthy of further study. In this review, we will discuss the functional roles of these genes/proteins, and hopefully, open new areas for AHPND research.

### 2.1. PirA^vp^ (ORF 51) and PirB^vp^ (ORF50)

The products of these two genes were named based on their sequence homology to a binary toxin family: *Photorhabdus* insect-related (Pir)-like proteins. As mentioned above, PirA*^vp^* and PirB*^vp^* have been confirmed as the major factors that cause damage to the hepatopancreatic tissues of shrimps, and this is considered the key symptom of AHPND [4,5,6,7,8,9,10]. Although some reports showed PirB*^vp^* alone has the ability to cause cell damage [5,6], both PirA*^vp^* and PirB*^vp^* are necessary to achieve full toxicity. The pirA*^vp^* and pirB*^vp^* genes are both part of the same operon [5], which means that they are regulated and expressed synchronously.

Structural analysis showed that there was a functional relationship between PirA*^vp^*/PirB*^vp^* and the *Bacillus thuringiensis* Cry toxin [5,9]. Functionally, Cry toxins act as pore-forming toxins and achieve cytotoxic effects via three domains: the *N*-terminal pore-forming domain I, the middle receptor binding domain II and *C*-terminal sugar/receptor binding domain III [43,44,45]. The typical cytotoxic mechanism of the Cry toxin is as follows: domain III first recognizes the *N*-Acetylgalactosamine (GalNAc) that is present on several receptors (e.g., aminopeptidase N, APN). Subsequently, domain II also binds to the recognized receptor. Then, the α1 helix of domain I is hydrolyzed, and this triggers the oligomerization of the Cry toxin to form pores on the cell membrane [43,44,45].

It was found that PirA*^vp^* and PirB*^vp^* have a Cry toxin-like folding [5,9]. The *N*-terminal and *C*-terminals domain of PirB*^vp^* (PirB*^vp^*N and PirB*^vp^*C) have a similar folding to the pore-forming domain I and receptor binding domain II of Cry toxin, respectively. The PirB*^vp^*N has an alpha-helical bundle structure, and this is called an inside-out membrane fold. The inside-out membrane fold consists of a hydrophobic α-helix surrounded by multiple amphipathic α-helices, and it is often found in other pore-forming toxins such as colicin and Cry toxins [9]. The structural characteristics of this domain allow it to switch between soluble (hydrophobic α-helix hides inside; inactivated) and transmembrane (hydrophobic α-helix exposed; activated and toxic) forms [9]. Meanwhile, the PirB*^vp^*C contains an antiparallel β-barrel jelly-roll topology. Despite the divergence of their amino acid sequences, PirB*^vp^*C and Cry domain II share a similar antiparallel β-barrel jelly-roll topology, suggesting that PirB*^vp^*C may also be involved in receptor binding. There is also a recent report which showed that alpha amylase-like protein, which is a 1,4-α-d-glucan glucanohydrolase targeted by the Cry toxin, has the ability to interact with PirB*^vp^* [46]. However, this result was based on far western and mass spectrometry analysis [46] and will need to be confirmed by other methods. Shrimp receptors for PirB*^vp^*C, thus still remain to be further investigated.

Like PirB*^vp^*C, PirA*^vp^* also has a jelly-roll topology. The structural analysis further shows that PirA*^vp^* has a substrate-binding pocket [9]. Although the precise targets of PirA*^vp^* have not yet been identified, the structural similarity of PirA*^vp^* to Cry domain III implies that these targets may include carbohydrates such as GalNAC [9].

**Figure 2:**
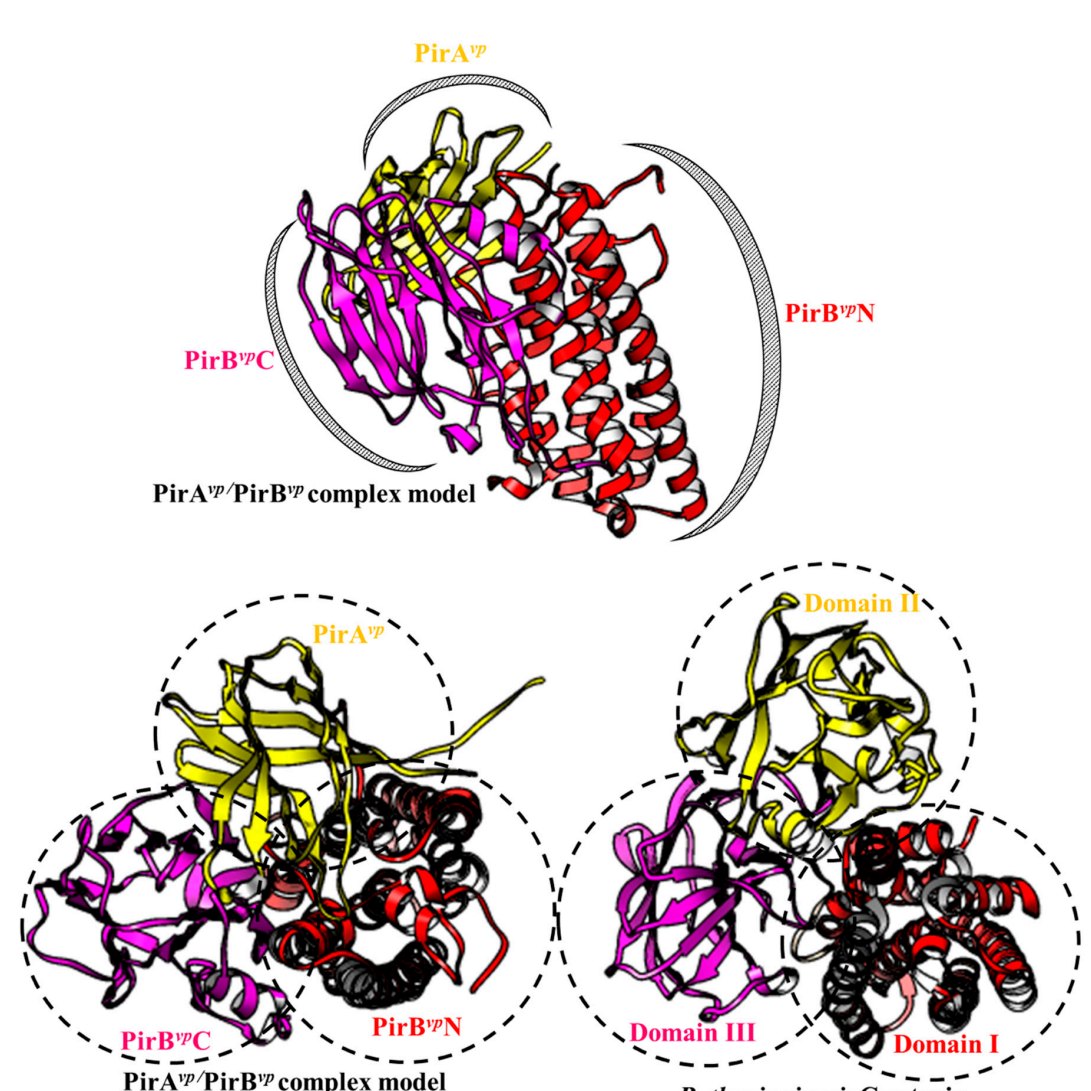
PirA*^vp^*/PirB*^vp^* binding model (**top**) and a comparison between the PirA*^vp^*/PirB*^vp^* binding model and the Cry toxins (**bottom**). The PirA*^vp^*/PirB*^vp^* binding model is based on our previous report [13]. The PDB (Protein Data Bank) number for Cry toxin is 1CIY.

Given their structural similarities, the cytotoxic activation of PirA*^vp^*/PirB*^vp^* should be similar to that of the Cry toxins. If so, then the first step for PirA*^vp^*/PirB*^vp^* would be to assemble into a Cry-like three-domain complex. In 2019, Lin et al. proposed binding models for PirA*^vp^*/PirB*^vp^* by using crosslinking based- and hydrogen-deuterium exchange-mass spectrometry. As shown in Figure 2, The PirA*^vp^*/PirB*^vp^*heterodimer model has all the functional domains that are necessary for its cytotoxicity: i.e., binding to the carbohydrate/receptor by PirA*^vp^* and PirB*^vp^*C would be followed by the creation of an uncontrollable pore by PirB*^vp^*N on the cell membrane to induce cell death. However, the binding affinity of PirA*^vp^* and PirB*^vp^* was found to be relatively low (~7 µM) in vitro [13], suggesting that PirA*^vp^* and PirB*^vp^* would not form a stable complex when the two proteins interact directly. It is possible, however, that the interaction with the receptor/sugar may improve the stability of the PirA*^vp^*/PirB*^vp^* complex. We also note that in contrast to the structural characteristics of PirA*^vp^* and PirB*^vp^*C discussed above, in this model, PirA*^vp^* and PirB*^vp^*C were matched respectively to the Cry receptor binding domain II and sugar-binding domain III (Figure 2 lower images). This suggests that the real functions of PirB*^vp^*C and PirA*^vp^* may be reversed. Interestingly, a recent report further supports this idea by finding that PirB*^vp^*, but not PirA*^vp^*, is able to target glycosaminoglycans like GalNH_2_ and GlcNH_2_ [47]. Clearly, more work needs to be done before we fully understand how these toxins, in fact, attach to the shrimp cell membrane.

### 2.2. Transposases (ORF15, ORF48, ORF55, ORF57 and ORF68)

DNA transposons are mobile DNA elements that can move from one DNA molecule to another. Transposases are a type of enzyme that can bind to the inverted sequences on both ends of a transposon, digest, and release the transposon, and promote translocation of this DNA fragment to another location on the genome [26,48]. This provides a mechanism to deliver genetic information into other chromosomes and confer new functions to a gene or replace a defective gene.

In pVA1, a total of five transposase genes were found (ORF15, 48, 55, 57, and 68). Apart from ORF57, which only contains a zinc-binding domain of the IS91 transposase family, the other four genes are complete transposases. ORF15, 48, and 55 share 100% sequence identity, and as members of the IS5 family of DDE transposases, they all contain a conserved DNA hydrolysis DDE (Asp-Asp-Glu) motif. The remaining transposase, ORF68, belongs to the Rpn family of recombination-promoting nuclease/putative transposases. All four of these transposases have the ability to trigger the transfer of DNA transposons.

In previous reports, it has been shown that the *pirAB^vp^* genes can be deleted/transferred by gene transposition. For instance, in the pVA1 plasmid isolated from *V. parahaemolyticus* strain 3HP, it was found that the ORF49-54 gene cluster, which includes the *pirAB^vp^* genes (ORF50 and ORF51) was flanked by two transposase genes in opposite directions (ORF 48 and ORF55) with inverted repeats in their terminals. It was also found that the gene cluster consisting of ORF 48-ORF54 was lost in the pVA1 type plasmid isolated from *V. parahaemolyticus* strain M2-36, leaving only ORF55 behind [5]. A gene cluster containing *pirAB^vp^* has also been inserted into another AHPND-causing plasmid, pVH, isolated from *V. owensii* [49]. The same transposase genes with inverted repeats were also found up- and down-stream of this cluster [49]. Another report describes a pVA1 plasmid from a natural *V. parahaemolyticus* strain (XN87), which contains a mutated *pirA^vp^* gene that is interrupted by a transposon gene fragment [50]. This results in an out-of-frame insertion of *pirA^vp^* and further affects the downstream gene expression of *pirB^vp^* [50]. Furthermore, whole-genome sequencing of 40 *V. parahaemolyticus* isolated from shrimp hepatopancreas and aquaculture water in Malaysia indicated that *pirAB^vp^* genes are prone to deletion [51]. Taken together, these results demonstrate the importance of this DNA transposon in the transfer, deletion, and mutation of the *pirAB^vp^*-containing gene cluster. Screening other bacteria or plasmids for the presence of this gene cluster will also be useful for identifying potential AHPND-causing pathogens.

### 2.3. Conjugation Factors (ORF10, ORF11, ORF75, ORF76, ORF78, ORF79, ORF81, ORF82, ORF83, and ORF84)

Conjugation is a process used by bacteria to transfer their genetic material to a recipient bacterium. This horizontal gene transfer facilitates bacterial evolution, including the dissemination of antibiotic resistance genes [52]. During conjugation, a single-stranded DNA molecule is first produced, and the plasmid undergoes DNA replication and formation of the protein-DNA complex, which is processed by the DNA transfer and replication (Dtr) system. The coupling protein (CP) further delivers this protein-DNA intermediate to the trans-membrane channel, where it is actively secreted through the channel and the exocellular pili, which are produced by the mating pair formation (Mpf) system. The Mpf/CP conjugation system is a kind of type-IV secretion system (T4SS), and there are twelve components essential for the Mpf system, TraF, TrbB, TrbC, TrbD, TrbE, TrbF, TrbG, TrbH, TrbI, TrbJ, TrbK, and TrbL [30,31]. On the pVA1 plasmid, all of these genes have been found except for *trbJ* and *trbK*. In addition, ORF11 is annotated as a VirB1-like gene, which is an important component that facilitates the formation of type-IV secretion systems (T4SS) [32]. This mechanism is, therefore, available for pVA1-related plasmids or genes to use for transferring between different bacterial cells.

Recently, conjugation inhibitors were considered as a potential means of preventing the spread of antibiotic resistance genes among bacteria [53]. For example, in the type-IV secretion system, bacterial conjugation could be inhibited by using unsaturated fatty acids to block VirB11′s activity [54]. Another report also showed that two compounds isolated from medicinal plants, rottlerin [5,7-dihydroxy-2,2-dimethyl-6-(2,4,6-trihydroxy-3-methyl-5-acetylbenzyl)-8-cinnamoyl-1,2-chromene] and the red compound (8-cinnamoyl-5,7-dihydroxy-2,2,6-trimethylchromene), both have the ability to inhibit conjugal transfer of plasmids pKM101, TP114, pUB307, and R6K among Gram-positive bacteria [55]. It may, therefore, be worth investigating if any compounds are able to block the pVA1-derived conjugation system. By preventing the spread of pVA1, the damage produced by AHPND might be limited.

### 2.4. Antirestriction Proteins (ORF32 and ORF35)

Among different species of bacteria, conjugative plasmids play a crucial role in spreading a variety of genes between various bacterial species. However, the transfer of DNA in naturally occurring vectors is tightly regulated by the immigration system, or restriction-modification (R-M) system, of the host [56]. The bacterial R-M system acts as an immune system that attacks the foreign DNA in the cell [56]. To overcome this restriction barrier of the host, some donor plasmids encode antirestriction proteins such as Ard (alleviation of restriction of DNA). On the pVA1 plasmid, there are two putative antirestriction protein genes, ORF32 and ORF35, which respectively have deduced amino acid sequences with similarity to ArdB and ArdC.

*Ard* genes are commonly found in transposons and conjugative plasmids in various prokaryotes [57]. The Ard antirestriction proteins are of three types, ArdA, ArdB, and ArdC, all of which act as inhibitors of the type I R-M system [33,34,58,59,60,61]. ArdA and ArdB proteins are small and acidic, and they both include a small region of similarity that consists of 14 residues designated as the “antirestriction” domain [58]. However, while ArdA has a DNA-like shape and charge distribution, suggesting it acts as a DNA-mimic [59,62], ArdB proteins have a novel structural fold, and do not show any DNA-like properties [60]. Meanwhile, ArdC proteins can also bind to single-stranded DNA, and they have been observed to protect single-stranded but not double-stranded plasmid DNA against the activity of type II restriction endonuclease (*Hha*I) in vitro [34]. In their role as type I R-M system inhibitors, ArdB and ArdC presumably act to prevent pVA1 from being digested during plasmid transmission. Conversely, if their anti-restriction activity can be blocked, the transferred pVA1 plasmid should then become susceptible to attack by the bacterium’s type I R-M system. This is an issue that deserves further investigation.

### 2.5. Secretion System (ORF3, 86, 89, and 90)

In addition to the type IV secretion system mentioned above (i.e., ORF11, VirB1-like gene), four other ORFs (ORF3, 86, 89, and 90) belonging to type II and III secretion systems are also found on the pVA1 plasmid. *V. parahaemolyticus* has been reported to contain not only two type-III secretion systems (T3SS1 and T3SS2) and two type-VI secretion systems (T6SS1 and T6SS2) [63,64], but also two T2SSs and two T2/4SSs [15]. These secretion systems are all related to the virulence of *V. parahaemolyticus*. For example, T3SS1 contributes to the secretion of effector proteins that are cytotoxic to HeLa cells, and T3SS2 is related to the enterotoxicity in the rabbit ileal loop test [65]. T6SS1 has been reported as an antibacterial system and is associated with the AHPND-causative strains [66]. In addition, T6SSs also contribute to the adhesion of *V. parahaemolyticus* to host cells [67], while T2SS is commonly employed by Gram-negative bacteria to transport a variety of molecular cargos [68]. Since PirA*^vp^* and PirB*^vp^* are both secreted toxins [5], the pVA1 secretion systems may play an important role during toxin release.

The secretion systems of drug-resistant bacteria were considered as the drug targets [69,70]. For example, *Acinetobacter baumannii* T2SS is responsible for the secretion of multiple enzymes, and its inactivation reduces the in vivo fitness of *A. baumannii,* as well as increasing its sensitivity to the human complementary system [70]. Recently, several small molecules, such as salicylidene acyl hydrazides and *N*-Hydroxybenzimidazoles, have been confirmed as T3SS inhibitors [69]. Moreover, high-throughput screening (HTS) based on secreted lipase activity was also developed to identify small molecule inhibitors of the T2SS [70]. Similarly, a search for inhibitors of the secretion systems of AHPND-causing *V. parahaemolyticus* might also identify molecules with the potential for controlling the damage caused by PirAB*^vp^* toxins.

### 2.6. pndA (ORF7)

A bacterial toxin-antitoxin system is a class of the plasmid addiction system (PAS) or post-segregational killing (PSK) system that is used to prevent the loss of plasmids from bacterial populations. For examples, in the *hok*-*sok* system of R1 plasmid, the *srnB-srnC* system of F plasmid and the *pndA-pndB* system of R438 plasmid, highly stable “killer” mRNAs that encode toxin proteins (Hok, SrnB, and PndA) are synthesized while small, unstable antisense RNAs (*sok, srnC* and *pndB*) are also produced. When coupled with host RNase II, these antisense RNAs facilitate the degradation of the killer mRNAs, and suppress the expression of toxin proteins [35]. However, after cell segregation, the production of antisense RNAs in the plasmid-free cells ceases, and due to their low stability, the levels of antisense RNAs rapidly decreases. Meanwhile, the killer mRNAs are still present. This results in the production of the encoded toxin proteins, each of which contains a single transmembrane domain, which is used to kill the plasmid-free cells from the inside [71,72]. The presence of a *pndA* gene (ORF7) in the pVA1 plasmid suggests that the PSK system is being used to ensure the presence of pVA1 in daughter cells. However, to date, the antisense RNA sequences for the *pndA* gene (i.e., *pndB*) have not been determined. Clearly, this will need to be further investigated to ascertain whether or not AHPND-causing bacteria actually include a viable pVA1-derived PSK system.

### 2.7. DNA Methyltransferase (ORF47 and ORF56)

DNA methylation is catalyzed by DNA methyltransferases (MTases, or methylases), which can transfer the methyl group from *S*-adenosyl methionine to the *C*-5 or *N*-4 positions of cytosine, or the *N*-6 position of adenine [73]. In bacteria, it is well known that DNA methylation protects the host’s own DNA from degradation by restriction-modification systems that are used to digest foreign DNA such as plasmids, transposons, and viral DNA [74,75]. In these cases, each MTase has its cognate restriction enzyme. However, some MTases (e.g., Dam and Dcm) do not have corresponding restriction enzymes, and instead of participating in restriction-modification systems, they are involved in the regulation of gene expression and virulence (for a review, please see [76]). ORF47 on the pVA1 plasmid is predicted to be an *N*-6 DNA MTase that is similar to Dam. Dam is essential for *Vibrio cholerae* virulence [36]. It also regulates the *finP* gene on the pSLT virulence plasmid of *S*almonella and affects the formation of F-type pilus that is responsible for plasmid transfer via conjugation [77]. It should, therefore, be worth investigating whether this predicted *N*-6 DNA MTase of pVA1 works like Dam, and further affects the virulence of the plasmid-harboring bacteria.

## 3. Conclusion Remarks

By incorporating the pVA1 plasmid, *Vibrio parahaemolyticus* becomes an AHPND-causing pathogen that is fatal to shrimps. Although the major virulence of the pVA1 plasmid is due to the PirA*^vp^*/PirB*^vp^* toxins, there are still ~40 important ORFs with functional annotations (Table 1). The ORFs discussed here are involved in toxin secretion, gene transposition, plasmid maintenance, bacterial conjugation, and a post-segregational killing (PSK) system. As discussed above, some gene products, like the conjugation factor and the secretion system, could be potential drug targets for combatting AHPND. Other genes, such as those for anti-restriction proteins, should also be worth investigating for their functional roles in the spread of the AHPND-causing plasmid among Vibrio spps. Taken together, we hope the information reviewed here will be useful for suggesting new research avenues for increasing our understanding of the mechanism of this disease.

## Figures and Tables

**Table 1:** The predicted open reading frames (ORFs) and their annotations.

**Pore Forming Toxins (2 ^†^)**				
**ORF**	**Gene/Protein Name**	Accession No. *	**Location and Direction**	**Function Annotation ^#^ and Related References**
ORF50	PirB*^vp^*	AKC05670	33699←35015	Photorhabdus insect-related (Pir) B; delta endotoxin, *N*-terminal domain [5,9]
ORF51	PirA*^vp^*	AKC05671	35028←35363	Photorhabdus insect-related (Pir) A [5,9]
**Transposition (5 ^†^)**				
**ORF**	**Gene/Protein Name**	Accession No. *	**Location and Direction**	**Function Annotation ^#^**
ORF15	Transposase	AKC05635	9224→10144	IS5 family transposase; DDE domain transposase [26]
ORF48	Transposase	AKC05668	32035←32955	IS5 family transposase; DDE domain transposase [26]
ORF55	Transposase	AKC05675	36541→37461	IS5 family transposase; DDE domain transposase [26]
ORF57	Transposase	AKC05677	38476→38607	Transposase zinc-binding domain; NCBI conserved domain: Zn_Tnp_IS91 (pfam14319) [27]
ORF68	Transposase	AKC05688	48532→49476	Rpn family recombination-promoting nuclease/putative transposase [28]
**Secretion System (4 ^†^)**				
**ORF**	**Gene/Protein Name**	Accession No. *	**Location and Direction**	**Function Annotation ^#^**
ORF3	Type IV pilin protein	AKC05623	1052→2011	Type II secretory pathway, pseudopilin PulG (Cell motility, Intracellular trafficking, secretion, and vesicular transport, Extracellular structures) [29]
ORF86	Type II and III secretion system protein	AKC05706	64495→65997	Pilus (MSHA, mannose-sensitive hemagglutinin type) biogenesis protein MshL; Bacterial type II and III secretion system protein [29]
ORF89	Type II secretion protein	AKC05709	67759→69381	Type II secretion protein; P-loop containing Nucleoside Triphosphate Hydrolases; Walker A motif; ATP binding site (chemical binding); Walker B motif [29]
ORF90	Type II secretion protein F	AKC05710	69378→70382	Type II secretion protein F [29]
**Conjugation Factor (11 ^†^)**				
**ORF**	**Gene/Protein Name**	Accession No. *	**Location and Direction**	**Function Annotation ^#^**
ORF10	TraF	AKC05630	5999→6511	Conjugative transfer signal peptidase TraF; Signal peptidase, peptidase S26 [30,31]
ORF11	VirB1-like conjugal transfer protein	AKC05631	6525→7040	Conjugal transfer protein; VirB1-like subfamily; one of twelve proteins making up type IV secretion systems (T4SS); VirB1 transglycosylase activity facilitate T4SS assembly [32]
ORF74	TrbC	AKC05694	54020→54349	Conjugal transfer protein TrbC; TrbC/VIRB2 family [30,31]
ORF75	TrbD	AKC05695	54360→54674	Mating pair formation protein TrbD; Type IV secretory pathway, VirB3-like protein [30,31]
ORF76	TrbE	AKC05696	54715→57129	Mating pair formation protein TrbE; conjugal transfer protein TrbE; Provisional; CagE, TrbE, VirB family, component of type IV transporter system; P-loop containing nucleoside triphosphate hydrolases [30,31]
ORF78	TrbB	AKC05698	57895→58842	Conjugal transfer protein TrbB; P-loop containing Nucleoside Triphosphate Hydrolases [30,31]
ORF79	TrbL	AKC05699	58839→60149	Conjugal transfer protein; TrbL/VirB6 plasmid conjugal transfer protein [30,31]
ORF81	TrbF	AKC05701	60380→61054	Conjugal transfer protein TrbF; VirB8 protein; putative dimerization motif (polypeptide binding) [30,31]
ORF82	TrbG	AKC05702	61063→61935	Conjugal transfer protein TrbG; VirB9/CagX/TrbG, a component of the type IV secretion system; VirB7 interaction site [30,31]
ORF83	TrbH	AKC05703	61935→62357	Conjugal transfer protein TrbH [30,31]
ORF84	TrbI	AKC05704	62350→63606	TrbI; conjugal transfer protein TrbI; Provisional; Bacterial conjugation TrbI-like protein [30,31]
**Antirestriction Protein (2 ^†^)**				
**ORF**	**Gene/Protein Name**	Accession No. *	**Location and Direction**	**Function Annotation ^#^**
ORF32	Antirestriction protein (ArdB)	AKC05652	21587←22051	Antirestriction protein; ArdB [33]
ORF35	DNA primase; antirestriction protein (ArdC)	AKC05655	22595←23488	DNA primase; antirestriction protein (ArdC) [34]
**Post-Segregational Killing System** **(1 ^†^)**				
**ORF**	**Gene/protein Name**	Accession No. *	**Location and Direction**	**Function Annotation ^#^**
ORF7	Protein hokC /pndA	AKC05627	4292→4444	Hok/gef family; Associated with a post-segregational killing (PSK) system [35]
**DNA methyltransferase (2 ^†^)**				
**ORF**	**Gene/Protein Name**	Accession No. *	**Location and Direction**	**Function Annotation ^#^**
ORF47	*N*-6 DNA Methylase family protein	AKC05667	30063←32042	*N*-6 DNA Methylase family protein [36]
ORF56	Methylase family protein	AKC05676	37446←37976	Methylase family protein [36]
**Known Function (15 ^†^)**				
**ORF**	**Gene/Protein Name**	Accession No. *	**Location and Direction**	**Function Annotation ^#^**
ORF5	Trypsin protease	AKC05625	2553→3638	Trypsin-like protease; NCBI conserved domain: Trypsin_2 (pfam13365) [27]
ORF21	DNA-binding protein	AKC05641	12652→13059	Domain in histone-like proteins of HNS family; NCBI conserved domain: Histone_HNS (pfam00816) [27]
ORF23	MobB	AKC05643	14053→16020	Molybdopterin-guanine dinucleotide biosynthesis protein MobB; P-loop containing nucleoside triphosphate hydrolases [37]
ORF26	Mobilization protein	AKC05646	16788→17528	Mobilization protein [37]
ORF27	DNA topoisomerase III	AKC05647	17595→19805	DNA topoisomerase III. DNA Topoisomerase, subtype IA; DNA-binding, ATP-binding and catalytic domain of bacterial DNA topoisomerases I and III, and eukaryotic DNA topoisomerase III and eubacterial and archael reverse gyrases [38]
ORF40	DNA-binding protein	AKC05660	24974←25270	Putative DNA-binding protein (no available reference)
ORF46	SSU ribosomal protein S2p	AKC05666	28045←29535	SSU (small subunit of ribosome) ribosomal protein S2p [39]
ORF53	Exported protein	AKC05673	35657←36133	Exported protein of unknown function [no available reference]
ORF61	Recombinase RecF	AKC05681	40846→43179	DNA repair exonuclease SbcCD ATPase subunit [Replication, recombination and repair] [40]
ORF62	Phosphatidylinositol kinase	AKC05682	43568→44392	Phosphatidylinositol kinase, CoiA-like protein [41]
ORF63	Cold-shock protein	AKC05683	44809←45483	Ribosomal protein S1-like RNA-binding domain. Found in a wide variety of RNA-associated proteins; NCBI conserved domain: CSP_CDS (cd04458) [27]
ORF64	Alpha-helical coiled coil protein	AKC05684	45537←46523	SMC_prok_B; chromosome segregation protein SMC, common bacterial type; NCBI conserved domains: KfrA_N (pfam11740) and CCDC158 (pfam15921) [27]
ORF66	Tyrosine-tRNA ligase	AKC05686	47258←47578	Putative tyrosine-tRNA ligase [no available reference]
ORF67	ParA	AKC05687	47562←48239	Chromosome partitioning protein ParA; plasmid-partitioning protein RepA [42]
ORF71	Rep	AKC05691	51712←52551	RNA polymerase Rpb2, domain 4; NCBI conserved domain: RNA_pol_Rpb2_4 (pfam04566) [27]

^†^ The number of each functional classification indicates how many pVA1 genes/proteins are classified into this type. *: Database source accession number: KP324996, #: Functional annotations were based on the NCBI Protein BLAST (blastp; https://blast.ncbi.nlm.nih.gov/Blast.cgi?PAGE=Proteins&). This analysis was achieved on February 2020.
